# Study on Analysis of Peripheral Biomarkers for Alzheimer’s Disease Diagnosis

**DOI:** 10.3389/fneur.2017.00328

**Published:** 2017-07-14

**Authors:** Palaniswamy Rani, Sreeram Krishnan, Chellappa Rani Cathrine

**Affiliations:** ^1^Department of Biotechnology, PSG College of Technology, Coimbatore, India

**Keywords:** Alzheimer’s disease, oxidative stress, biomarkers, redox status, lipid peroxidation

## Abstract

Many factors are involved in Alzheimer’s disease (AD) pathology including tau phosphorylation, amyloid β protein (Aβ) accumulation, lipid dysregulation, oxidative stress, and inflammation. The markers of these pathological processes in cerebral spinal fluid are used currently for AD diagnosis. However, peripheral biomarkers are the need of the hour for large population screening for AD. The main objective of the present study is to evaluate the peripheral levels of redox markers, lipid peroxidation (LPO) indicators, and pathological markers in AD patients. Blood was collected from AD patients (*n* = 45), controls (*n* = 45), and analyzed for pathological markers of AD including Aβ42 and tau, LPO, and redox indicators. Plasma Aβ42 was significantly (*P* < 0.001) elevated while total tau was decreased in AD compared to controls. Hydroxynonenal (HNE) and malondialdehyde (MDA) were higher (*P* < 0.001) in AD patients pointing the enhanced LPO in AD pathology. Receiver operating characteristic curve (ROC) analysis indicated that HNE is a better indicator of LPO compared to MDA. Plasma glutathione (GSH) level was significantly (*P* < 0.001) low while oxidized glutathione (GSSG) level was higher (*P* < 0.001) in AD patients with corresponding decrease in GSH/GSSG ratio (*P* < 0.001). ROC analysis indicated that GSH/GSSG ratio can be used as reliable indicator for redox imbalance in AD with a cutoff value of <8.73 (sensitivity 91.1%, specificity 97.8%). Correlation analysis revealed a positive correlation for both HNE and MDA with Aβ42 and a negative correlation with total tau. Negative correlation was observed between GSH/GSSG ratio and LPO markers. While oxidative stress has been implicated in pathology of various neurodegenerative disorders, the present study pinpoints the direct link between LPO and Aβ production in plasma of AD patients. Normally, at low amyloid concentration in body fluids, this peptide shown to function as a strong metal chelating antioxidant. However, when the Aβ production enhanced as in AD, through gain of functional transformation, Aβ evolves into prooxidant, thereby enhancing oxidative stress and LPO. Altered redox status with enhanced LPO observed in AD blood could contribute to the oxidation and *S*-glutathionylation proteins, which has to be addressed in future studies.

## Introduction

Alzheimer’s disease (AD) is a debilitating illness of the nervous system, affecting millions of elderly individuals worldwide. Numerous factors are involved in the disease etiology including tau phosphorylation, amyloid β protein (Aβ) accumulation, lipid dysregulation, oxidative stress, and inflammation. Among all factors, oxidative stress plays a central role in the pathogenesis of AD leading to neuronal dysfunction and cell death (1). Oxidative stress is caused by increased production of reactive oxygen species (ROS), which denature biomolecules such as proteins, lipids, and nucleic acids through pathological redox reactions (2). Increased oxidative stress also results in excessive lipid peroxidation (LPO), which in turn perturb the bilayer structure and modify the membrane properties such as membrane fluidity, permeability, and bilayer thickness (3). This might cause an imbalance in the concentration of ions and impairing metabolism, thereby leading to apoptosis and necrosis. Oxidative stress has been emphasized in pathology of various inflammatory diseases including cancer, atherosclerosis, diabetes, and neurodegenerative disorders (4). However, human brain is more vulnerable to oxidative stress due to high oxygen consumption and high concentration of easily oxidizable polyunsaturated fatty acid. Particularly, LPO markers are found to be elevated in brain tissue and fluid in several neurodegenerative disorders. The status of LPO has extensively analyzed in AD, PD. There are also controversy regarding the LPO marker in neurodegenerative disorder, whether it represents the distinct pathomechanism of the specific disease or merely a non-specific occurrence (5).

The human body is enriched with many endogenous antioxidants to scavenge excess ROS produced. Antioxidant enzymes like peroxidase, superoxide dismutase along with glutathione (GSH), help in nullifying the ill effects of excess ROS. While the levels of these antioxidants are shown to be elevated in AD brain owing to neuronal damage, increase in the levels of peripheral antioxidants observed indicate that the damage is not limited to brain alone (6, 7). Alteration in levels of circulating antioxidants is also directly related to the severity of cognitive impairment (8). Elevated levels of markers of LPO including hydroxynonenal (HNE) and malondialdehyde (MDA) were reported in AD (9, 10).

Reduced glutathione (GSH), in parallel with oxidized glutathione (GSSG), is generally responsible for maintaining redox balance of the cell. Mandal et al. (11) reported a reduction in GSH levels in the brain of AD patients along with an alteration in GSH/GSSG ratio, indicating excessive oxidative damage. Moreover, incidence of increased protein glutathionylation has also been reported in the inferior parietal lobule of AD brain (12). Estimating the markers of LPO and redox status over a period of time would be helpful in assessing the disease progression. Since AD is a brain-related disease, only a little evidence is present on the peripheral manifestation. Research on identifying the blood-related biomarkers of AD is in fast progress since the determination of biomarkers in cerebral spinal fluid (CSF) is invasive, expensive, and time-consuming. Although amyloid beta 1–42 (Aβ42) and total tau in blood are being currently considered for diagnosis of AD (13), there is a need to determine other peripheral markers of AD because of the multiple pathological processes involved. Thus, the present study is designed with a principal objective to assess the peripheral LPO and redox markers in AD, and correlating their levels with the pathological markers in peripheral blood in AD patients. The study was focused on identification of a panel of oxidative stress markers in blood along with Aβ42 and tau for diagnosis of AD.

## Materials and Methods

### Study Population

The present study was approved by the Institutional Ethical Committee of PSG Institute of Medical Sciences and Research (PSG IMS&R), Madras Medical College and Government General Hospital (MMC), Chennai, and all procedures involving human subject complied with the Declaration of Helsinki. Informed consent was obtained from control subject and from the relatives, legal guardians of the AD patients before their inclusion in the study. The AD patients (*n* = 45) involved in the study were recruited from MMC, Chennai, PSG IMS&R, Coimbatore, and Alzheimer’s and Related Disorders Society of India (ARDSI), Thrissur, India. The cognitive status of the subject was assessed using Mini-Mental State Examination (MMSE) (14), and categorized according to the criteria of NINCDS-ADRDA and DSM-IV for dementia (15). The AD patients with cardiovascular disease and diabetes were excluded in the study. The control subject (*n* = 45) included in the study were in the age group of 50–70 years with a negative history of cardiovascular disorders, diabetes, and neurological disorders. Subject with a habit of smoking, alcohol consumption, or exhibiting comorbidity with advanced malignancies, AIDS, and other major illness were excluded from the study.

### Sample Collection

Blood samples (10 mL) were collected from each study subject in EDTA tubes and plasma was separated by centrifugation at 2,500–3,000 rpm for 15 min. The separated plasma samples were aliquoted in microfuge tubes (0.5 mL) and stored at −20°C for further use.

### Pathological Markers of AD

Aβ42 and total tau levels in plasma were quantified using a commercially available ELISA kit from CUSABIO, China (CSB-E10684h, CSB-E12011h). The sandwich ELISA procedure was employed in these kits and the yellow color developed because of horse radish peroxidase reaction was read at 450 nm with a wavelength correction of 540 nm in a plate reader.

### LPO Markers

Lipid peroxidation markers including HNE and MDA were evaluated in the present study. The assay of MDA was performed by thiobarbituric acid (TBA) assay method described by Buege and Aust (16). The reaction of MDA with TBA formed a red-colored MDA–TBA complex, which was measured at 535 nm. HNE was determined in plasma samples as described by Benedetti et al. (17). The method involves the carbonyl derivatization of samples with 2,4-dinitrophenylhydrazine, thin-layer chromatographic separation of 2,4-dinitrophenylhydrazine carbonyls, and HPLC-based analysis of dinitrophenylhydrazone of HNE, which was detected at 378 nm.

### Redox Markers

The oxidized and reduced glutathione was measured using the colorimetric method of analysis (18). Reduced glutathione (GSH) reacts with Ellman’s Reagent [5,5′-dithio-bis-(2-nitrobenzoic acid)] DTNB to generate 2-nitro 5-thiobenzoic acid, which was determined by measuring absorbance at 412 nm. The assay of oxidized glutathione (GSSG) is based on the glutathione recycling system using glutathione reductase (GR) and DTNB (19).

### Statistical Analysis

The results were expressed as a mean ± SD. Statistical analysis was performed using the statistical package SPSS Version.16, Chicago, IL, USA. All parameters were under the normal distribution, except for the amyloid and tau, for which Mann–Whitney *U*-test was performed to compare between AD and control. Receiver operating characteristic (ROC) curve analysis was performed to study the reliability of HNE, MDA, and GSH/GSSG ratio in plasma as markers for AD diagnosis. Pearson’s correlation was done wherever applicable.

## Results

The products of pathophysiological processes in AD brain are shown to diffuse to the blood (20). This study focuses on analysis of such products in plasma of AD patients, which could lead to the identification of reliable blood-based biomarkers, which would serve to reflect the underlying AD pathology.

The AD patients and controls involved in the study were in the age group of 60–80 years. The mean age of AD patients is 71.73 ± 8.84 years and the control is 67.46 ± 8.29. Assessment of the cognitive status of the subject indicated that AD patients exhibited profound to severe loss in their cognitive abilities (Table 1).

**Table 1:** Cognitive status of Study group.

Subject (*n* = 45)	Mean age (years)	Mean MMSE
Alzheimer’s disease (male = 24; female = 21)	71.73 ± 8.84	3.50 ± 2.5
Controls (male = 22; female = 23)	67.46 ± 8.29	28.15 ± 2.60

*Values are expressed as mean ± SD*.

*Mini Mental State Examination (MMSE) score used for measuring the degree of cognitive impairment with the following scale*.

*0–4, →profound; 4–13, →severe; 13–16, →moderate; 17–23, →mild; 24–30, →normal*.

### Pathological Markers of AD

The CSF tau and Aβ42 are the biochemical markers currently used in AD diagnosis (21). Since diagnosis could mainly rely on plasma markers, the plasma levels of pathological markers of AD, including total tau and Aβ42 were evaluated. The plasma Aβ42 level was found to be twofold higher in AD compared to control. Whereas total tau was found to be decreased by 1.8-fold in AD patients. The increased Aβ42 levels with decreased tau resulted in corresponding decrease in tau–amyloid ratio in AD patients compared to control group and this variation was statistically significant (*P* < 0.001) (Table 2). There was no significant association observed between pathological markers and MMSE score (Amyloid *r* = −0.054; *P* > 0.05 and total tau *r* = −0.085; *P* > 0.05).

**Table 2:** Plasma levels of amyloid, tau, and tau/amyloid ratio.

Subject	Amyloid, pg/ml	Tau, pg/ml	Tau/amyloid ratio
Alzheimer’s disease	174.87 ± 62.15	451.76 ± 240.82	3.08 ± 2.35
Control	90.62 ± 42.35	836.93 ± 369.31	13.36 ± 4.42

*The values are articulated as mean ± SD*.

### Peripheral LPO Markers in AD

**Figure 1:**
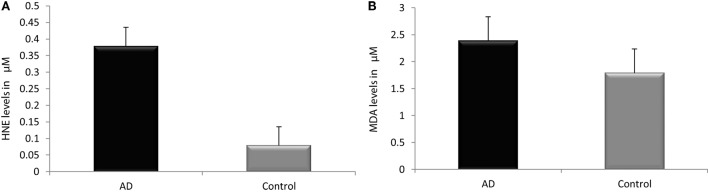
Assessment of lipid peroxidation markers. **(A)** Hydroxynonenal, **(B)** malondialdehyde in plasma of Alzheimer’s disease and control. Data represents the mean ± SD. *P < 0.001, significantly different from control group.

**Figure 2:**
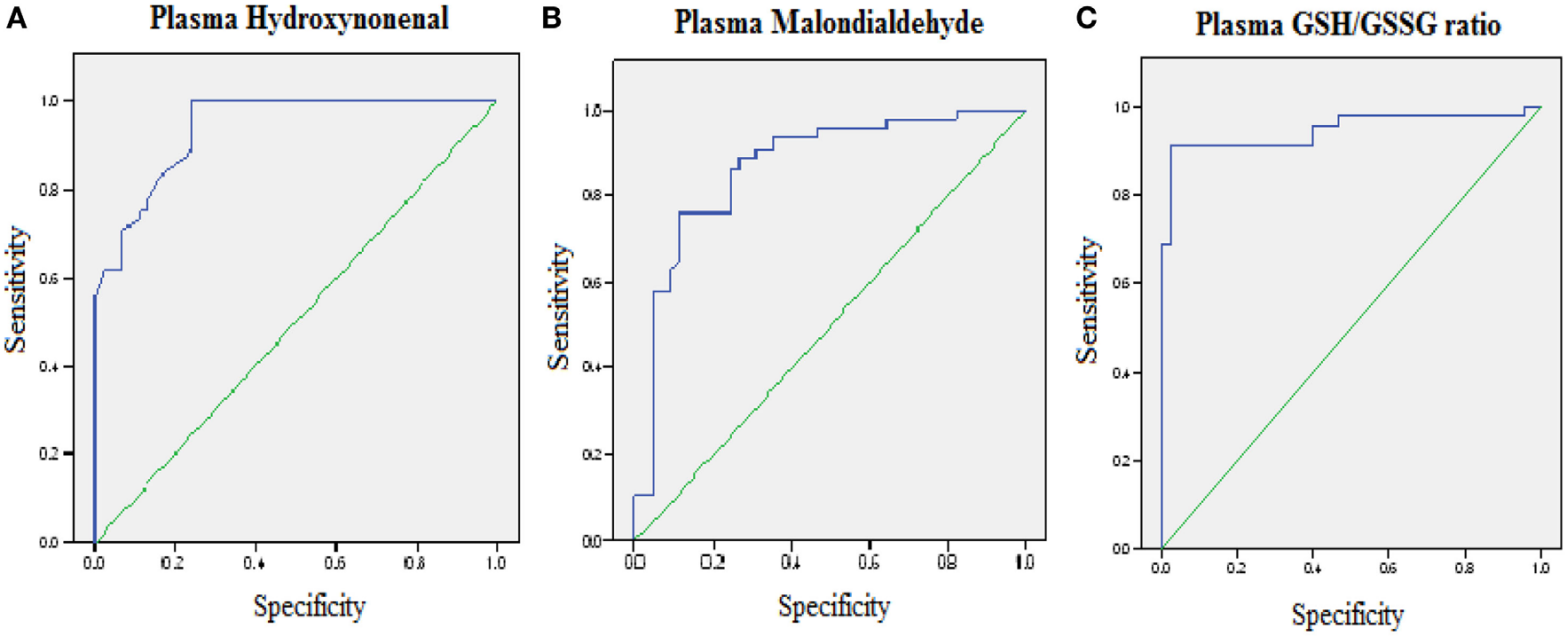
Receiver operating characteristic curve analysis. **(A)** Hydroxynonenal—area under the curve (AUC): 0.93 (*P* < 0.01), cutoff: >0.1057 µM, sensitivity: 93.5%, specificity: 75%; **(B)** malondialdehyde—AUC: 0.87 (*P* < 0.01), cutoff: >1.98 µM, sensitivity: 80%, specificity: 75%; **(C)** GSH/GSSG ratio—AUC: 0.95 (*P* < 0.01), cutoff: <8.73, sensitivity: 91.1%, specificity: 97.8%. The AUC determines the quality of diagnostic test: 0.9–1 excellent, 0.8– 0.9 good, 0.7–0.8 fair, 0.6–0.7 poor, 0.5–0.6 fail.

**Figure 3:**
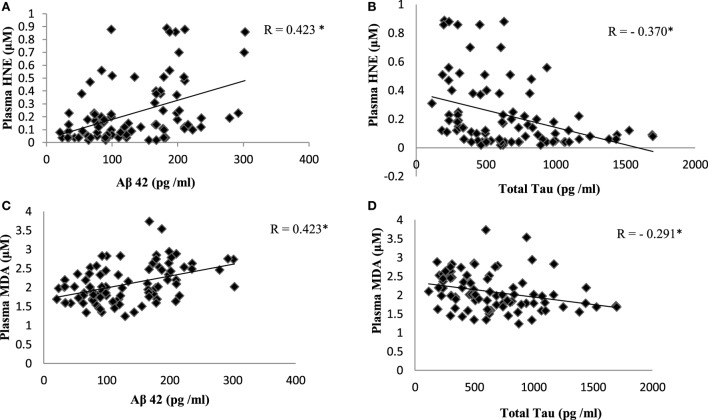
Correlation analysis between plasma lipid peroxidation markers and pathological markers of Alzheimer’s disease: **(A)** hydroxynonenal (HNE) and Aβ42; **(B)** HNE and total tau; **(C)** malondialdehyde (MDA) and Aβ42; **(D)** MDA and total tau. Correlation is significant at *P* < 0.01.

Reactive-free radicals alter phospholipids structure and function in cell membranes leading to the formation of lipid peroxides, generating the various small compounds namely MDA and HNE. These two compounds are assessed routinely as indicators of LPO index. In the present study, HNE and MDA were analyzed in plasma of AD patients to assess the oxidative damage in the cellular system. Significant increase in HNE (0.38 ± 0.26 µM) level was observed in plasma of AD patients compare to control group (0.08 ± 0.05 µM; *P* < 0.001). The MDA level (2.38 ± 0.44 μM) was also found increased in plasma samples of AD patients (1.78 ± 0.33 μM; *P* < 0.001). The results indicated that both HNE and MDA were elevated in AD patients pointing the enhanced LPO in AD (Figure 1). ROC analysis was performed to validate the use of both HNE and MDA as plasma markers of AD. It was observed that HNE was a better indicator of LPO in AD with a cut-off value of >0.105 μM (sensitivity 93.5%, specificity 75%) compared to MDA (cutoff: >1.98 μM, sensitivity 80%, specificity 75%) (Figure 2). Among the plasma pathological markers of AD, Aβ42 peptide showed positive correlation with HNE (*r* = 0.423; *P* < 0.01) and MDA (*r* = 0.423; *P* < 0.01) whereas negative correlation was observed for total tau with HNE (*r* = −0.370; *P* < 0.01) and MDA (*r* = −0.291; *P* < 0.01) (Figure 3). However, no significant correlation was observed between MMSE and LPO markers including HNE (*r* = 0.184; *P* > 0.05) MDA (*r* = 0.128; *P* > 0.05).

### Plasma Redox Markers in AD

**Figure 4:**
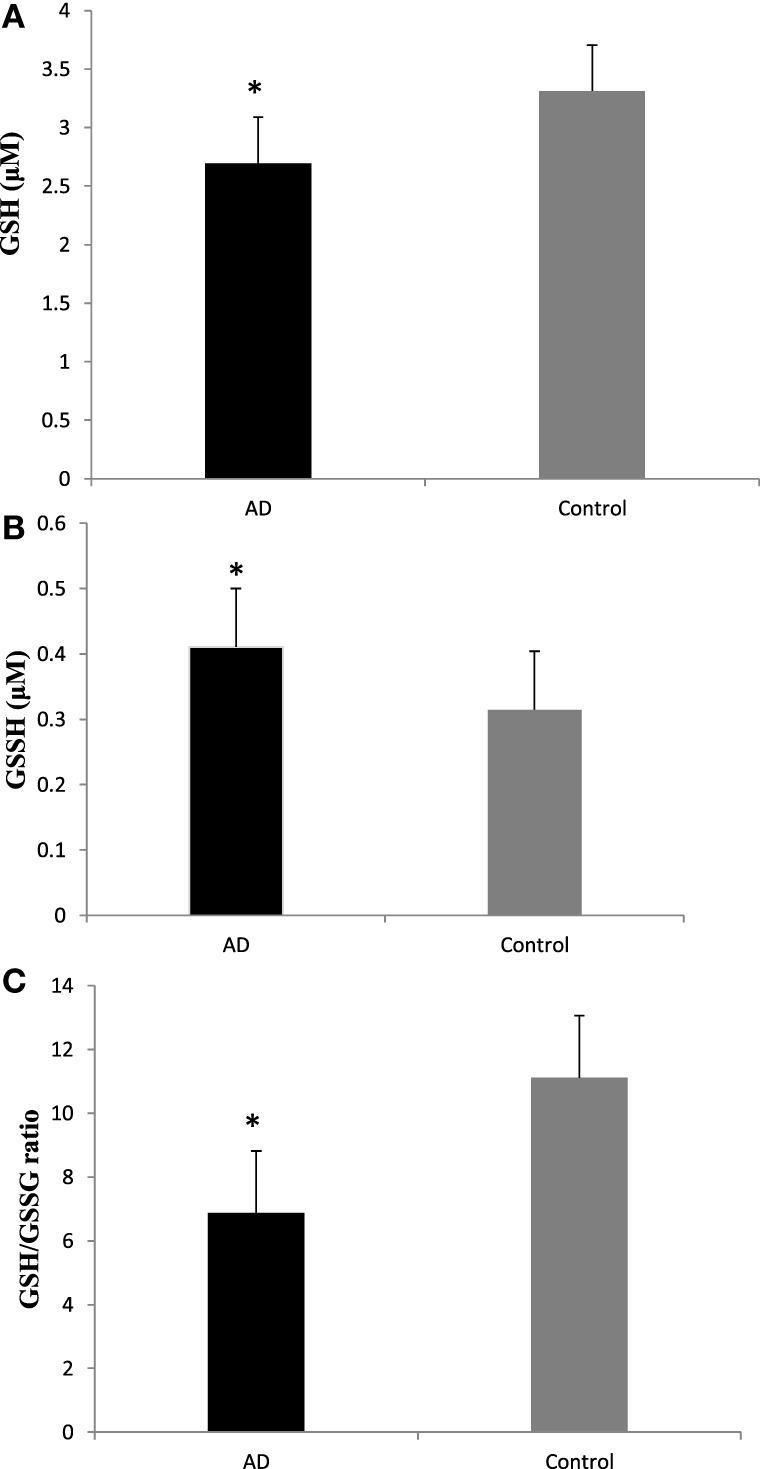
Plasma redox status in Alzheimer’s disease patients **(A)** GSH, **(B)**, GSSG **(C)**, and GSH/GSSG ratio. Data represents the mean ± SD. *P < 0.001, significantly different from control group.

**Figure 5:**
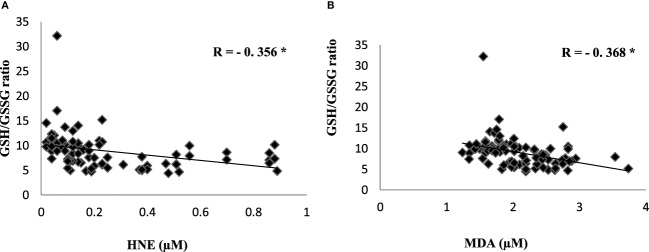
Correlation analysis of redox markers with lipid peroxidation markers in Alzheimer’s disease. **(A)** HNE, **(B)** malondialdehyde. Correlation is significant at *P* < 0.001.

**Figure 6:**
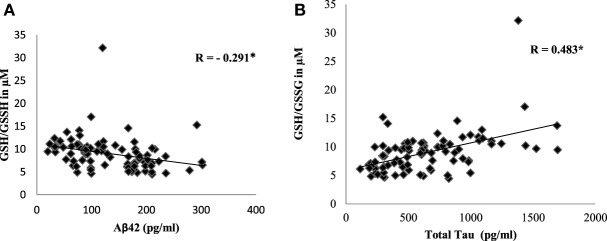
Correlation analysis of GSH/GSSH ratio with pathological markers in Alzheimer’s disease. **(A)** Aβ42, **(B)** total tau. Correlation is significant at *P* < 0.001.

To evaluate the redox status of the peripheral system, GSH, GSSG, and GSH/GSSG ratio were analyzed in the plasma of AD patients. The results revealed that GSH was significantly reduced by 84% (*P* < 0.001) while GSSG was increased by 74% (*P* < 0.001) in AD patients compared to controls contributing to the corresponding decrease of about 54% in GSH/GSSG ratio (*P* < 0.001) correspondingly (Figure 4). The changes observed in the redox status of the blood were correlated with increased LPO markers. Negative correlation was observed between GSH/GSSG ratio and LPO markers HNE (*r* = −0.356; *P* < 0.01) MDA (*r* = −0.368; *P* < 0.01) (Figure 5). GSH/GSSG ratio also correlated with AD pathological markers namely Aβ42 (*r* = −0.291; *P* < 0.01) and total tau (*r* = 0.483; *P* < 0.01) (Figure 6). ROC analysis indicated that GSH/GSSG ratio can be used as reliable marker for the diagnosis of AD with a cutoff value of <8.73 (sensitivity 91.1%, specificity 97.8%) (Figure 2). The correlation analysis between redox markers and MMSE has shown no significant relationship (GSH *r* = −0.215; *P* > 0.05; GSSG *r* = −0.148; *P* > 0.05 and GSH/GSSG ratio *r* = 0.002; *P* > 0.05).

## Discussion

Oxidative stress plays a pivotal role in driving the pathological process of AD. While the symptoms of AD appear later in life, the oxidative damage to the neurons starts decades, before the onset of the actual symptoms (6). Since timely diagnosis is imperative to monitor the progression of the disease and to prevent further damage, identifying reliable peripheral markers are of utmost importance. In the present study, we have evaluated the LPO indicators: HNE and MDA, redox peptides: GSH and GSSG, in order to validate their use as potential oxidative stress markers of AD by correlating their levels with known pathological markers namely Aβ42 and total tau in AD blood samples.

The well-known pathological markers of Alzheimer’s disease (AD) are extracellular amyloid beta peptide (Aβ peptide) plaques and intracellular neurofibrillary tangles (NFTs) of hyperphosphorylated Tau (22). Elevated levels of tau, phosphorylated tau, and amyloid peptide were reported in CSF of AD patients suggesting their use as markers for clinical trials (23). However, contradictory reports exist regarding their levels in blood (24–26). Similar plasma amyloid (Aβ42) and total tau levels were also reported by Pesini et al. (27) and Sparks et al. (25) respectively. To facilitate less invasive diagnosis, peripheral blood markers are more useful than CSF markers and other expensive brain imaging techniques. Among different peripheral tissues, blood reflects the major physiological changes in various body organs and systems including brain (28).

The results of the present study indicate that plasma Aβ42 was increased and total tau was decreased in AD patients compared to control group with the corresponding decrease in the tau-to-amyloid ratio. Aβ in plasma could be influenced by the transport of Aβ peptide across blood–brain barrier (BBB) (29). Aβ peptide in the brain is cleared to the systemic circulation through LRP1 a low density lipoprotein receptor related protein in BBB. However transport of Aβ peptide into the brain is processed through receptor for advanced glycation end products RAGE (30). Hence the synergistic expression of LRP and RAGE could govern the transport of Aβ peptide across the BBB.

Apart from brain, the tissues like skeletal muscles, platelets, kidney, pancreas, spleen and liver also contribute to the peripheral pool of Aβ peptides. This limits the use of plasma Aβ42 as a specific marker for AD. Neurofibrillary tangle is a hallmark of AD which occurs mainly due to abnormal phosphorylation of tau proteins. Several studies reported the elevated levels of CSF tau (31). However few reports exist regarding tau concentration in blood (32). Plog et al (33) indicated that during traumatic brain injury, tau can reach the blood via the glymphatic system. In the present study total tau level was decreased in plasma of AD patients compared to age- matched control group. Yet a meta- analysis study indicated that total tau in plasma alone might not be a reliable marker for diagnosis (13) and mechanistic view of tau in the peripheral pool is not well understood. However the decrease in plasma tau could be attributed due to the defective clearance of tau from the CNS to blood in AD patients. As a consequence, excess tau builds up in the CNS leading to the formation of NFTs. Sparks et al. (25) also reported a decline in cognitive function with decreased plasma total tau levels. Although contradictory reports exist regarding Aβ and tau independently as plasma markers, the result of our present study indicated that the tau-to-amyloid ratio may be used as an effective marker for AD diagnosis (13).

Lipid peroxidation is a process, wherein the free radicals attack the lipid components resulting in the generation of lipid peroxides and hydroperoxides (34) leading to molecular cellular damage and augmenting many pathological conditions including aging. LPO results in the formation of an aldehyde such as MDA, HNE, which are of great consideration because of their high reactivity and toxicity to the biological compounds of the cell (35). Traditionally, MDA is used as a convenient biomarker for LPO but due to the non-specificity of its measurement, it is less reliable (36). However, HNE is reported as a secondary messenger of free radicals and also a major generator of oxidative stress (37). Nearly 1–8% of the HNE production in a cell is known to target the proteins (38).

Lipid peroxidation is also known to cause damage to the neuronal membrane (28) resulting in the formation of toxic secondary products, which are responsible for further cellular damage because of their ability to diffuse from the site of formation (39). Oxidative stress had been majorly implicated in AD pathogenesis and Aβ is shown to be the major source of oxidative stress in CNS (35). When neurons were treated with Aβ, it resulted in the formation of HNE (40). Further, when NT2 neurons were exposed to HNE, enhanced production of Aβ was observed due to increase in activity of γ secretase and β secretase (41). These studies emphasis the role of oxidative stress in amyloid synthesis leading to AD. Exact role of LPO in AD pathology is unclear, however, increased production of amyloid as a metal chelating antioxidant could result in pathological aggregation of amyloid due to its interaction with metal ions, thereby enhancing oxidative stress leading to oxidation of lipid and other biomolecules, thereby enhancing neurotoxicity (42).

Hydroxynonenal is known to form protein adducts, which get deposited in the various region of the brain and is also shown to diffuse across BBB, thereby forming protein adducts in body fluids like CSF and blood (43). Elevated levels of HNE and MDA were reported in the brain (10, 44) and in erythrocytes of AD patients (1, 9–11). The present study also indicates increased levels of HNE and MDA in the blood of AD patients compared to age-matched controls. ROC analysis also revealed that HNE could be an effective marker for AD diagnosis. Studies indicated that the abnormal small vessel structure mainly affecting the endothelium occurs in AD leading to abnormal BBB function (45). This might have contributed to the elevated levels of blood HNE in AD patients by the transport of HNE from brain to blood. Positive correlation observed between plasma HNE levels and AD pathological markers exemplifies the same.

GSH plays a fundamental role in detoxification of ROS and also maintains intracellular redox environment of the cell effectively. GSH is also reported as a neuroprotectant by nullifying the toxic effect of ROS in neuronal cells (46, 47). Depletion of GSH observed in the brain of AD patients and also in AD animal models indicates the protective role of GSH in the age-related neurodegenerative disorders. A balance between GSH and GSSG levels are maintained by the enzymes glutathione peroxidase (GPx) and GR. Gpx scavenges the excess ROS produced as a result of excessive oxidative stress by oxidizing GSH to GSSG. This is reversed by GR, thereby maintaining the levels of GSH in the cells. Numerous studies have reported an increase in the activity of GPx and decreased GR activity in AD patients, thereby altering the levels of GSH (8, 9). The reduction in the levels of GSH with an increase in GSSG observed in blood samples of AD patients is attributed mainly due to the alteration in the activity of GPx and GR as reported earlier in our study (9). A decrease in GSH/GSSG ratio was also observed in both blood and brain of AD mice (48). The results of the present study indicates pertubance in redox status and alteration in pathological markers in plasma of AD patients; however, these markers are not associated with MMSE scores since plasma levels of these constituents are also influenced by other peripheral tissue sources, thereby limiting their use as specific diagnostic marker for AD.

Taking consideration of oxidative damage in AD pathology, many studies have focused on use of antioxidants as potential therapeutics. Most antioxidant drugs established general success in animal models, however, shown less beneficial effects in human trials; clinical trials for AD prevention by antioxidants supplements are still in initial stage and further studies are quite necessary to determine if antioxidants may decrease the risk or slow the progression of the disease for AD patients (49).

Alteration in redox homeostasis leads to the modification of protein like oxidation and *S*-glutathionylation. *S*-glutathionylated actin was reported in AD brain by Dalle-Donne et al. (50). Other proteins such as GAPDH, hemoglobin, crystalline B, α enolase also shown to be thiolated, causing a disturbance in glucose metabolism and oxygen supply in neurons (12, 51). In the present study, alteration in redox status along with enhanced LPO observed in the blood of AD patients might also contribute to thiolation of crucial blood proteins. Further studies are warranted to investigate the exact mechanism behind the protein thiolation due to elevated LPO.

## Ethics Statement

This study was carried out in accordance with the recommendations from Institutional Ethical Committee, PSG Institute of Medical Sciences and Research (PSG IMS&R), Coimbatore, and Madras Medical College and Government General Hospital (MMC), Chennai with written informed consent from all subjects. All subjects gave written informed consent in accordance with the Declaration of Helsinki. The protocol was approved by the Institutional Ethical Committee of PSG IMSR and MMC.

## Author Contributions

PR, SK, RC: conception and design of study; acquisition of data; analysis and/or interpretation of data; drafting the manuscript; revising the manuscript critically for important intellectual content; approval of the version of the manuscript to be published.

## Conflict of Interest Statement

The authors declare that the research was conducted in the absence of any commercial or financial relationships that could be construed as a potential conflict of interest.
